# An In Vivo Microfluidic Study of Bacterial Load Dynamics and Absorption in the *C. elegans* Intestine

**DOI:** 10.3390/mi12070832

**Published:** 2021-07-17

**Authors:** Vittorio Viri, Maël Arveiler, Thomas Lehnert, Martin A. M. Gijs

**Affiliations:** Laboratory of Microsystems, Ecole Polytechnique Fédérale de Lausanne, CH-1015 Lausanne, Switzerland; vittorio.viri@gmail.com (V.V.); mael.arveiler@chimieparistech.psl.eu (M.A.); thomas.lehnert@epfl.ch (T.L.)

**Keywords:** *C. elegans*, microfluidics, bacteria, absorption

## Abstract

*Caenorhabditiselegans* (*C. elegans*) has gained importance as a model for studying host-microbiota interactions and bacterial infections related to human pathogens. Assessing the fate of ingested bacteria in the worm’s intestine is therefore of great interest, in particular with respect to normal bacterial digestion or intestinal colonization by pathogens. Here, we report an in vivo study of bacteria in the gut of *C. elegans*. We take advantage of a polydimethylsiloxane (PDMS) microfluidic device enabling passive immobilization of adult worms under physiological conditions. Non-pathogenic *Escherichia coli* (*E. coli*) bacteria expressing either pH-sensitive or pH-insensitive fluorescence reporters as well as fluorescently marked indigestible microbeads were used for the different assays. Dynamic fluorescence patterns of the bacterial load in the worm gut were conveniently monitored by time-lapse imaging. Cyclic motion of the bacterial load due to peristaltic activity of the gut was observed and biochemical digestion of *E. coli* was characterized by high-resolution fluorescence imaging of the worm’s intestine. We could discriminate between individual intact bacteria and diffuse signals related to disrupted bacteria that can be digested. From the decay of the diffuse fluorescent signal, we determined a digestion time constant of 14 ± 4 s. In order to evaluate the possibility to perform infection assays with our platform, immobilized *C. elegans* worms were fed pathogenic *Mycobacterium marinum* (*M. marinum*) bacteria. We analyzed bacterial fate and accumulation in the gut of N2 worms and mitochondrial stress response in a *hsp-6::gfp* mutant.

## 1. Introduction

The millimeter-sized roundworm *C. elegans* is an established model for many fields in biomedical research. Due to genetic resemblance and amenability, *C. elegans* is highly relevant to human biology, e.g., for the study of neurodegenerative diseases or aging [1,2,3,4]. Moreover, the availability of a wide range of mutants and human disease models makes *C. elegans* a versatile tool for next-generation high-content screening platforms [5]. Bacteria are the worms’ natural food source and worms develop a proper gut microbiome in their natural environment [6], whereas *E. coli* is used as the main food source in laboratory settings. In order to develop and sustain its physiological metabolism, a *C. elegans* worm needs to consume a considerable amount of bacteria over its lifespan, requiring highly efficient food processing and digestion mechanisms. Ingestion and pharyngeal transport of bacterial food are fast processes that have been monitored by high-speed video imaging using microparticles, for instance [7]. Advanced methods, including bacterial clearing assays, pulse-feeding assays with nitrogen isotope labeled bacteria, or automated bioluminescence-based measurements, have also been used for food intake and nutrient absorption studies [8,9,10]. As an example, Ding et al. used a bioluminescent *E. coli* strain seeded on nematode growth medium (NGM) plates to quantify foraging and feeding of *C. elegans* N2 wild-type worms, *eat-2* mutants presenting a deficiency in pharyngeal pumping, and *npr-1* mutants [10]. As bioluminescence is coupled to living bacteria, food uptake could be quantified by the loss of signal after ingestion and digestion. After initial mechanical disruption of bacterial food in the pharyngeal grinder, small bacteria fragments were shown to transit into the gut for enzymatic digestion [11], where lysozymes and other enzymes may further break down membrane fragments and their lipid constituents [12]. Digestive hydrolase in *C. elegans*, including protease activity, relies on a finely regulated acidic environment in the gut [13,14]. The pH variation in the *C. elegans* gut, which was determined by pH-sensitive fluorescence nanosensors, was found to range from 5.96 ± 0.31 in the anterior pharynx to 3.59 ± 0.09 in the posterior intestine [15]. Moreover, dynamic processes in the acidification profile of the intestinal lumen could be mapped in real-time and different oscillatory pH patterns have been reported [15,16,17]. For instance, during the defecation motor program, a spot of higher acidity rapidly moves periodically from the posterior to the anterior intestine every 45–50 s, with temporary localization for several seconds in the anterior region [17].

In recent years, *C. elegans* has become increasingly interesting as a model for a systems-level understanding of microbiota-host interactions [18]. Assays are generally based on assessing the worm’s life/healthspan for different bacterial strains, gene expression analysis, or by evaluating intestinal bacterial proliferation [19,20,21,22]. For instance, Stuhr et al. explored how the most commonly used *E. coli* strains and bacterial diet genera found in the natural environment of *C. elegans* affect multiple life history traits of the worms [22]. Interestingly, *C. elegans* is also a relevant model for human infectious disease research [23,24,25]. A range of tactics that pathogenic microbes apply to injure *C. elegans* has been identified, in many cases related to pathogen accumulation in the intestine [23,26]. For instance, *Microbacterium nematophilum* was found to induce a distinctive swollen tail full of bacteria [27,28]. Likewise, *Salmonella typhimurium* proliferates and fills the worm intestine with intact bacteria within 48 h [25]. On the other hand, beneficial bacteria or probiotics can prolong *C. elegans* survival. For instance, Donato et al. demonstrated that *Bacillus subtilis* biofilm germination in the worm intestine extends longevity of nematodes by down-regulation of the insulin-like signaling (ILS) pathway [29]. Moreover, pathogens can induce dysfunction of the physiological mitochondrial activity of *C. elegans* [30,31], which leads to activation of mitochondrial repair, drug detoxification, and pathogen response pathways [32,33]. For instance, worm exposure to *Pseudomonas aeruginosa* induces mitochondrial dysfunction and the activation of the mitochondrial unfolded protein response (UPR^mt^) in *C. elegans* [34,35].

In this context, advanced methods and protocols for monitoring the properties and dynamics of the bacterial load in the *C. elegans* intestine emerge as an important requirement to directly analyze specific features of the microbial–host interaction and the resulting physiological or morphological impact. Taking advantage of microfluidic approaches could be very beneficial to explore high-content digestion or infection studies, based on accurate high-resolution imaging of the temporal and spatial evolution of the bacterial load. Contrary to most conventional methods, integrated on-chip arrays enabling enhanced assay versatility and accuracy, along with automated high-resolution time-lapse image recording and increased throughput, can actually be easily implemented within a microfluidic system. In particular, a microfluidic-based approach for *C. elegans* culturing brings significant advantages in terms of control of worm exposure to bacterial food. Fluidic protocols for worm feeding on-chip can be optimized to maintain a uniform and constant amount of bacterial suspension available for worms, or, on the other hand, more complex time-dependent feeding protocols can be envisioned.

However, suitable microfluidic devices addressing related requirements, for instance long-term immobilization under physiological conditions, are not often used and optimized protocols still need to be implemented. Different approaches for on-chip worm immobilization have been discussed previously. Trapping by confinement in tapered microfluidic channels was one of the earliest techniques and is now used for different applications, including large-array imaging platforms [36,37,38,39]. Immobilization based on active elements, in particular pneumatic valves on PDMS chips, has been successfully used for high-resolution worm imaging, however, this option increases the complexity of the device, hinders easy access to food, and exerts significant pressure on the worm body [40]. Kopito et al. developed a microfluidic system (WormSpa) where worms were confined individually in elongated ergonomic chambers [41]. The authors showed that confined worms maintained normal physiological functions and were not stressed over periods of 24 h and longer. Importantly, worms were capable to feed and to lay embryos during immobilization. More recently, Berger et al. presented a device for long-term immobilization of normally developing *C. elegans*, where worms are isolated by a set of on-chip valves in dedicated channel sections that perfectly match the worm body. The concept was validated by assessment of anchor cell invasion and distal tip cell migration in larval *C. elegans*, and germ cell apoptosis in adult *C. elegans* [42]. For the time being, only very few microfluidic worm infection assays have been proposed. In general they focus on killing assays and gene expression, but not on imaging protocols of the bacterial load in the worm gut [43,44,45,46].

Here, we present innovative dynamic imaging protocols of bacteria in the *C. elegans* intestinal track. We take advantage of a PDMS device comprising distinct microfluidic structures for worm culturing and long-term immobilization under physiological conditions. We monitored the transit of ingested bacteria, the spatial distribution and the dynamics of the intestinal bacterial load due to peristaltic activity over 30 h. Furthermore, we demonstrate that our microfluidic device is suitable for high-resolution imaging of fluorescently marked single bacteria persisting in the worm gut. Accurate discrimination between signals originating from intact and disrupted *E. coli* bacteria allowed determining the time constant of nutrient absorption in the gut during biochemical digestion. We further validated our method by monitoring the expression of the *hsp-6::gfp* stress reporter in *C. elegans* mutants exposed to pathogenic *M. marinum*.

## 2. Materials and Methods

### 2.1. Materials and Chemicals

4-inch 550 μm thick Si wafers and 5-inch Cr/quartz glass masks were obtained from the EPFL Center of MicroNanoTechnology (Lausanne, Switzerland). SU-8 3050 negative epoxy photoresist was purchased from micro resist technology GmbH (Berlin, Germany). PDMS Sylgard 184 was acquired from Dow Silicones Deutschland GmbH (Wiesbaden, Germany). Microline ethyl vinyl acetate tube (0.51 mm inner and 1.52 mm outer diameter) was bought from Fisher Scientific AG (Wohlen, Switzerland). Corning^®^ (75 mm × 50 mm, thickness 960 μm) microscope slides were purchased from Sigma-Aldrich Chemie GmbH (Buchs, Switzerland). S-medium for worm culture was prepared with 1 L of S Basal, 10 mL 1 M potassium citrate (pH 6), 10 mL trace metals solution, 3 mL 1 M CaCl_2_ and 3 mL 1 M MgSO_4_. Lysogeny Broth (LB) for *E. coli* bacteria liquid culture, tetracycline, ampicillin and kanamycin, Virkon^®^ and 1 μm-size rhodamine B marked melamine resin microparticles were purchased from Sigma-Aldrich (Buchs, Switzerland). Middlebrook 7H9 broth for *M. marinum* culture was obtained from the EPFL Laboratory of Microbiology and Microtechnology (EPFL-LMIC, Lausanne, Switzerland).

### 2.2. C. elegans Culture and Bacterial Sample Preparation

*C. elegans* N2 worms, DA465 *eat-2*(*ad465*) II, and SJ4100 *zcIs13[hsp-6::gfp]* mutants were provided by the Caenorhabditis Genetics Center (University of Minnesota). In order to obtain synchronized *C. elegans* populations, embryos were extracted from gravid adult worms by a standard bleaching protocol and cultured at 20 °C on nematode growth media (NGM) agar plates seeded with *E. coli* strain OP50. At young adult (YA) stage, worms were collected from the agar plates, suspended in S-medium and loaded into the microfluidic device. All experiments on-chip were performed at 20 °C in a temperature controlled environment. Different bacterial samples were used as food source for the on-chip assays. *E. coli* OP50 expressing red fluorescent protein (RFP) was constructed by transformation with plasmid vector *pRZT3::dsRED* using standard methods. pRZT3 also contains a sequence for tetracycline resistance. Unlabeled *E. coli* OP50 were grown in LB and *E. coli* OP50 RFP in LB with 25 μg/mL tetracycline. The GFP expressing *E. coli* HT115 strain (RRID:WB-STRAIN:HT115) was inoculated in LB with 100 μg/mL ampicillin and 10 μg/mL kanamycin. All *E. coli* bacteria were cultured overnight in a shaker at 37 °C. *M. marinum* bacteria (unlabeled or GFP labeled) were inoculated in Middlebrook 7H9 broth and grown for 2 days at 30 °C. After culture, bacterial suspensions were centrifuged, then bacteria pellets were resuspended in filtered S-medium and diluted to a concentration of 4 × 10^9^ cells/mL. Bacterial samples were freshly prepared for each experiment and the concentration was measured prior to each experiment with a WPA CO 8000 Biowave Cell Density Meter (Biochrom Ltd., Cambourne, UK). *E. coli* OP50 and HT115 GFP strains were provided by the EPFL Laboratory of Integrative Systems Physiology (EPFL-LISP) and *E. coli* OP50 RFP by the *C. elegans* Ageing Laboratory (University College London). *M. marinum* strains were provided by the EPFL Laboratory of Microbiology and Microtechnology (EPFL-LMIC).

### 2.3. Fabrication of the Microfluidic Chips

Microfluidic devices were prepared by soft lithography using SU-8 molds on a Si wafer for casting the PDMS. Conventional photolithography was used to pattern first a SU-8 20 μm-thick layer and subsequently a 40 μm-thick layer on top in order to fabricate a microfluidic device with different structure heights. SU-8 molds were coated with trimethylsilyl chloride (TMCS) to prevent PDMS sticking during PDMS demolding. Then, a liquid PDMS mixture (10:1 base to crosslinker weight ratio) was degassed, poured on the SU-8 mold and cured at 80 °C for 2 h. After demolding and hole punching, the PDMS chip was air plasma activated, bonded onto a glass slide, and connected to external tubing.

### 2.4. Statistical Analysis

Statistical analysis and curve fitting were performed using Graph Pad Prism software (San Diego, CA, USA, RRID = SCR_002798). For each experiment, *n* indicates the number of viable worms analyzed. All worms that died from matricidal hatching were excluded. Statistical significance was determined with a two-tailed Mann–Whitney test.

## 3. Experimental

### 3.1. Microfluidic Chip Design

**Figure 1:**
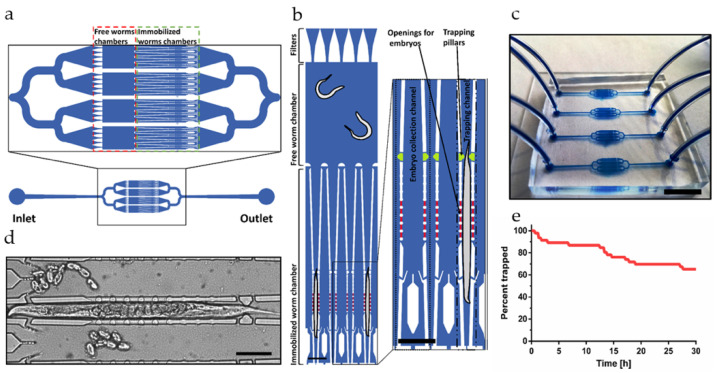
Microfluidic device for *C. elegans* immobilization and intestinal bacterial load imaging. (**a**) Schematic representation of an on-chip assay unit with 4 large channels each comprising a free-worm chamber (850 µm × 1200 µm, 60 µm high) and a section with 4 worm traps. (**b**) Schematic view of one of the channels. Passive PDMS filter structures (upper part of the figure) are located in front of each free-worm chamber to allow selective loading of YA worms. Smaller tapered channels guide the worms into the traps. Each trap has 2 opposite passive PDMS pillar structures for secured worm confinement (highlighted in green). Evacuation of embryos is possible through an arrangement of openings (20 µm × 25 µm, 40 µm high, highlighted in red) in the sidewalls of the traps. Feeding of immobilized worms independently from the body orientation is enabled by 2 lateral openings close to the end of the trap (see enlarged schematic view) and lateral openings at the location of the 2 trapping pillar structures (green). Scale bar = 200 μm. (**c**) Photograph of the PDMS chip with tubing, featuring parallel units for 4 independent assays. Scale bar = 10 mm. (**d**) Brightfield image of a N2 adult *C. elegans* worm immobilized with head-first position in one of the traps. Embryos laid by the worm were moving from the trap into the adjacent embryo collection compartments. Scale bar = 100 μm. (**e**) Percentage of trapped worms measured over 30 h (*n* = 46).

The bacterial digestion and colonization assays presented in this work take advantage of a monolithic PDMS microfluidic device comprising distinct adjacent microfluidic chambers for worm culturing and worm immobilization of *C. elegans*. The design for the microfluidic worm traps of our chip has been inspired by the WormSpa proposed by Kopito et al. that proved to provide physiological conditions for worm development during long-term (24 h) confinement [41]. For the assays discussed in the present work, stress-free confinement in an ergonomic environment is of particular importance, for instance, to maintain normal peristaltic activity of the worm gut. Figure S1a compares pharyngeal pumping rates measured in N2 YA worms on-chip for both free-swimming and trapped conditions, and on a conventional agar plate culture. Immobilization did not significantly affect the eating behavior of the worms. In our device, we introduced additional design features that enabled worm immobilization merely by passive elements without application of continuous flow. A microfluidic assay unit on the chip comprises 4 larger parallel channels (60 μm high) with 2 distinct compartments for culturing and immobilization of up to 4 individual worms (Figure 1a). YA worms were loaded via the chip inlet and pushed through dedicated on-chip PDMS filters into the culture chambers. For immobilization, worms were relocated and guided via small tapered channels into the on-chip traps. Figure 1b shows the 2-compartement arrangement and an enlarged schematic view with detailed features of the worm trap array. A picture of the whole microfluidic chip is shown in Figure 1c, comprising 4 independent units for assay parallelization, i.e., for testing of 4 different experimental conditions independently. Most important features of the worm traps are: (i) 2 passive PDMS trapping pillar structures for secured worm immobilization (highlighted in green in Figure 1b), (ii) an arrangement of side openings (highlighted in red in Figure 1b) for evacuation of embryos into adjacent collection compartments, and (iii) additional lateral openings for adequate worm feeding. To initiate immobilization, the nematode was transferred into the trap and pushed through the constriction formed by the 2 opposite PDMS pillar structures at the entrance of each trap. In this way, trapped worms could be confined for up to 30 h without application of continuous flow. Further to minimizing worm movement, the design of the trapping channels focused on preventing bagging, i.e., internal embryo hatching due to prolonged compression of the worm body. For this purpose, the sidewalls of the microtraps were accommodated with an array of pillars and apertures (40 μm high openings) through which eggs laid during worm immobilization could naturally pass into dedicated collections chambers. A small aperture at the end of these collection channels allowed flushing of embryos and L1 larvae to the chip outlet. Figure 1d shows a brightfield image of a confined gravid adult worm. Eggs can be seen inside the worm body, whereas laid eggs are in the adjacent embryo collection compartments. Possible starving during worm immobilization also had to be considered. Due to the impossibility of controlling the orientation of a worm in the trapping channel, the fluidic design had to be adapted to allow worm feeding in either position and to guarantee a proper amount of suspended bacteria around the mouth region. In Figure 1d, the worm was trapped in a head-first position. In this case, specifically shaped openings generate a backflow and redirect bacterial food injected through the embryo collection compartments towards the worm’s head (Figure 1b,d). In case of tail-first trapping, bacterial food was supplied through openings adjacent to the two trapping pillars close to the entrance of each trap.

### 3.2. Operation of the Microfluidic Platform

Automated protocols for all fluidic operations were implemented. Before an experiment, each unit of the microfluidic device was flushed with 1 mL of 70% ethanol for sterilization, rinsed and then filled with S-medium. For worm loading, chip inlets were connected to a dedicated reservoir containing an age-synchronized population of about 30 YA *C. elegans* suspended in S-medium. A pulsed flow sequence was applied for loading the YA worms into a microfluidic unit, thus distributing equally 7 or 8 worms into each on-chip free-worm culture chamber through the branched secondary channels (Figure 1a). During this operation the YA worms need to be pushed through the filter lines delimiting the culture chambers (Figure 1b). These V-shaped filters facilitate the passage of the worms during loading and prevent them from escaping when the applied flow is reversed. The filter pore size (minimum width 30 μm) was adjusted for YA worms. Subsequently, a second pulsed flow sequence was applied for worm immobilization until most of the traps of a unit were hosting a single worm. The fluidic protocol for worm trapping consisted of two steps. First, worms were gently pushed toward the entrance sections of the trapping channels close to the constriction defined by pillars (green in Figure 1b) by application of a low flow rate (1 μL/s) for 4 s. Subsequently, a fluidic pulse with higher flow rate (20 μL/s) was applied for 0.5 s in order to push the worms through the trapping pillars and secure them in the traps. In order to maximize the number of worms trapped in the device, the 2-step fluidic protocol was repeated 8 to 10 times without interruption.

At the end of this process, each unit contained approximately 4 × (3–4) immobilized worms, whereas typically 3 to 4 freely moving worms remained in each of the 4 culture chambers. Subsequently, the worm reservoirs were replaced by reservoirs containing bacterial suspensions for worm feeding. Flow pulses were only applied for worm loading, worm transfer for trapping, and for replenishment of the chip with fresh bacteria. Otherwise, worms remained confined in the traps without fluidic control. Worms confined in traps were allowed to slightly move longitudinally, i.e., along the trapping channel axis. The maximum displacement from the initial trapping position was about ± 30 µm over 24 h (*n* = 10). Figure 1e indicates that good long-term trapping efficiency was achieved, with 70% of the worms still being trapped after 30 h.

For experiments involving non-pathogenic *E. coli*, each microfluidic unit on the chip was connected to a computer-controlled V6 Kloehn syringe pump (Norgren Inc., Littleton, CO, USA) via a rotary valve for selectively actuating feeding protocols of parallel assays. An equivalent procedure was applied for experiments involving pathogenic *M. marinum* bacteria. In this case, dedicated 1 mL disposable syringes mounted on a computer-controlled neMESYS syringe pump (CETONI GmbH, Korbußen, Germany) were used for worm loading and feeding protocols.

This setup allowed simplifying the management of contaminated material and minimized the risk of cross-contamination. Bacterial suspensions were injected every 10 min for worm feeding. The time interval between two consecutive injections was selected in order to keep the amount of bacteria in the chambers approximately constant. The bacterial solution was automatically stirred by magnetic stirrers before injection to avoid sedimentation in the syringe reservoir thus improving the control of the amount of food dispensed to the worms. Using fluorescent bacteria as a food source allowed easy verification of the food availability around the head region of the immobilized worms in the traps. The injected bacterial sample volume per pulse was 3 µL. Such low volumes efficiently reduce the risk of bacterial agglomerate formation and consequently of channel clogging, which often compromises the functioning of PDMS microfluidic tools.

### 3.3. Imaging Protocol of the Worm Intestine

The microfluidic device was mounted on an upright microscope (Zeiss Axio Imager 2) equipped with a sCMOS pco.edge 4.2 LT camera (PCO AG, Kelheim, Germany) and two illumination systems for brightfield and fluorescence imaging, respectively. The motorized computer-controlled xyz-stage (Visitron Systems GmbH, Puchheim, Germany) enabled automated time-lapse image acquisition of the entire worm trap array. VisiView^®^ imaging software (Visitron, Germany) was used for this purpose. Image processing was done with ImageJ 1.51 n software (https://imagej.nih.gov/ij/. Accessed on 1 February 2019) and Matlab R 2106b software (MathWorks, Natick, MA, USA) was used to process data of the intestinal bacterial load in an automated manner. We performed long-term imaging, up to 30 h, for monitoring bacteria accumulation in N2 worms or *eat-2* mutants and of *hsp-6::gfp* expression in the corresponding mutants, respectively. Shorter time-lapse imaging sequences of the gut (up to 180 s) were recorded for assessing bacterial transit, peristaltic motion, and the fluorescence decay due to biochemical digestion. For long-term imaging, brightfield and fluorescence images were recorded successively but quasi-simultaneously, whereas for the short sequences with higher time resolution both images were acquired simultaneously with double-light exposure (i.e., white/545 nm for RFP imaging and white/488 nm for GFP imaging, respectively). Simultaneous acquisition of brightfield/fluorescent images was necessary in order to precisely locate the position of the fluorescent signal of the bacterial load with respect to anatomical structures of the worm. Double-light exposure did not affect the signal originating from fluorescently labeled *E. coli* (Figure S2). Depending on the specific assay, low-resolution (using a Zeiss LD Plan-NEOFLUAR 20× NA 0.4 objective) and/or high-resolution imaging (using a Zeiss 50× NA 0.55 objective) was performed. We demonstrated that the autofluorescence signal originating from a worm’s intestine is negligible with respect to the signal generated by fluorescently labeled *E. coli* bacteria (Figure S3).

## 4. Results

### 4.1. Monitoring of the Bacterial Load in the C. elegans Intestine

**Figure 2:**
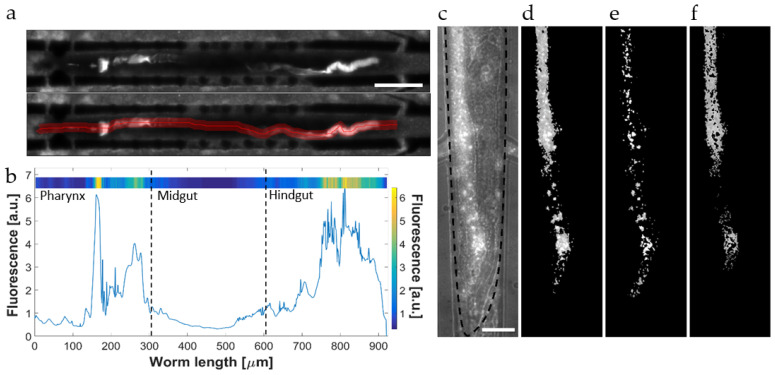
Automated image analysis for fluorescence measurements of the bacterial load in the worm’s gastrointestinal tract. (**a**) Low-magnification (20×) fluorescent image of a trapped adult N2 worm fed *E. coli* OP50 expressing RFP. The fluorescent signal generated by the bacterial load is mainly visible in the pharyngeal (grinder) and the hindgut regions. The signal was measured along a manually defined line corresponding to the intestinal tract (averaged over a width of 20 µm, lower image). Scale bar = 100 µm. (**b**) Fluorescence amplitude measured over the whole worm length from the head (*l* = 0 µm) to the tail region (*l* = 920 µm). For clarity, the intestine was segmented in three parts, indicated as pharynx, midgut, and hindgut. These regions are delimited by dashed lines in the plot. The peak at *l* = 150 µm and the broader peak centered at *l* = 800 µm correspond to the bright regions in (**a**). A linear color code scheme of the graph was generated (panel in the upper part of Figure 2b). (**c**) High-resolution (50×) brightfield/fluorescence imaging (hindgut of a N2 adult worm, highlighted by the dashed line) reveals diffuse fluorescence in the gut generated by disrupted bacteria and fluorescent spots related to the presence of intact bacteria/bacterial clusters. (**d**) The total fluorescence distribution and (**e**) bright signals related to intact bacteria are discriminated by applying two subsequent thresholding steps. (**f**) The signal associated with diffuse fluorescence is obtained by subtraction of (**e**) from (**d**). Scale bar = 25 µm.

**Figure 3:**
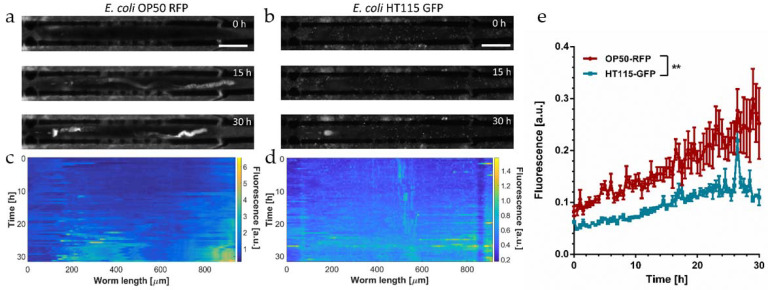
Analysis of the bacterial load in immobilized adult N2 *C. elegans* worms fed fluorescently marked *E. coli* bacteria. Fluorophores with different pH sensitivity were used to label the bacterial food. (**a**) Time-lapse sequence of fluorescence images (20×) over 30 h of a N2 worm fed *E. coli* OP50 RFP (pH-insensitive), and (**b**) of a N2 worm fed *E. coli* HT115 GFP (pH-sensitive). In the aging worms, an increase in fluorescence in distinct sections of the gut is observed, in particular in the pharyngeal region (for OP50 RFP and HT115 GFP), and in the hindgut (only for OP50 RFP). The strong RFP signal in the posterior region of the intestine is due to the presence of a mixture of intact bacteria and disrupted bacteria. Unlike HT115 GFP, both signals persist due to RFP pH insensitivity. (**c**,**d**) Color-coded maps of the fluorescence intensity measured over the whole worm gut, representing the evolution of the bacterial load over 30 h, for the worms fed OP50 RFP (**c**) and HT115 GFP (**d**), respectively. (**e**) Total fluorescence averaged over the entire worm gut over 30 h for both cases. A general increase over time of the bacterial load is observed, which is particularly evident in worms fed the OP50 RFP strain. In HT115 GFP-fed worms, the overall intensity of fluorescence is lower due to the acidic environment of the hindgut. Values have been normalized with respect to the maximum fluorescence in the pharynx occurring for an event of maximum bacterial intake. (** *p* ≤ 0.1, *n* = 10 for each condition). Error bars correspond to mean ± SD. Scale bars = 100 μm.

Worms mechanically disrupt ingested bacterial food in the pharyngeal grinder. Subsequently, peristaltic action transports bacterial fragments and nutrients into the mid and posterior regions of the gastrointestinal tract for chemical metabolization during the digestive process and finally residues are expelled by activation of a defecation motor program [47]. Expulsion of undigested material takes place approximately every 45–50 s for N2 worms [48]. We have used low-magnification (20×) imaging for monitoring the bacterial load of the entire gut of immobilized adult N2 worms. A representative example illustrating our custom-developed image analysis protocol is shown in Figure 2a [49]. In this specific case of a worm fed *E. coli* OP50 RFP (adult N2 worm, trapped for 30 h, see Figure 3a), the fluorescent image revealed enhanced bacterial load in the terminal bulb (grinder) of the pharynx, which is separated from the intestine by the pharyngeal-intestine valve, and in the posterior region of the intestine (hindgut) (Figure 2a, upper image) [50].

For quantification of bacterial accumulation, the fluorescent signal was measured along a manually defined line following the intestinal profile (Figure 2a, lower image). Mean values were calculated over a width of 25 µm and represented graphically by plotting the fluorescence intensity as a function of the location in the worm gut (Figure 2b).

The graph was then transposed into a linear color code scheme for more convenient visualization of the fluorescence distribution (panel in the upper part of Figure 2b). For monitoring the dynamics of bacterial/microbead transit or accumulation, an adequate number of such color plots was recorded successively over the time interval of interest and grouped into larger panels.

Figure 2c shows a high-resolution brightfield/fluorescent image (50×) of the hindgut of an adult worm, allowing to locate precisely the bacterial load (*E. coli* OP50 RFP). As shown in this example, we generally observed a superposition of diffuse fluorescence distributed in the lumen and brighter spots of small and varying size. As bacteria are mechanically disrupted in situ in the worm’s grinder, we assume that the diffuse signal may be attributed mainly to the presence of active fluorophores originating from membrane fragments and compounds from the cytoplasm [51], i.e., nutritive compounds that can be biochemically transformed in the worm intestine. In the present context, quantification of the diffuse fluorescent signal is therefore important for evaluating the digestive process. On the other hand, depending on the efficacy of the grinding process, also a certain amount of intact single bacteria and bacterial clusters (identified as the brighter spots in Figure 2c) can be found in locations posterior to the grinder.

In order to study the specific features and the fate of the bacterial load in more detail, we therefore designed an imaging protocol for discriminating between diffuse and discrete fluorescent signals in the intestinal track. We discriminated in vitro intact and disrupted fluorescently labeled bacteria by a simple brightness level thresholding protocol (Figure S4). For bacteria detection in worms’ gut, two subsequent thresholding steps were applied for extracting the total fluorescent signal intensity *I*_t_ (Figure 2d) and the brighter fluorescent spots *I*_i_ corresponding to intact bacteria that persist in the intestine (Figure 2e). The signal *I*_d_ associated with diffuse fluorescence of disrupted bacteria was obtained by subtraction *I*_d_ = *I*_t_ − *I*_i_ (Figure 2f). As will be reported later (see Figure 6e,f), this image processing protocol was particularly useful for evaluating the time constant *τ* of biochemical digestion based on the decay of *I*_d_.

The acidic environment in the *C. elegans* intestine, in particular in the mid and posterior sections, may alter signals generated by the fluorescently labeled bacterial load [15]. For this reason, and in particular for a correct and detailed interpretation of the presence, specific features and time dependence of the bacterial load, the pH sensitivity of the fluorophore expressed by the bacteria has to be considered. In a previous work, we characterized in vitro the pH sensitivity of two different *E. coli* strains, OP50 RFP and HT115 GFP, expressing RFP or GFP fluorescent proteins, respectively. We showed that the RFP marker was not affected in the pH range between 3.7 and 6, while the GFP intensity rapidly decreased even in mildly acidic solutions (pH 6) [49]. Here we studied the fate of these two *E. coli* strains in vivo. YA N2 nematodes were loaded in the worm microfluidic traps and identical feeding protocols were applied, either using *E. coli* OP50 RFP or HT115 GFP in different assay units of the chip. An example for each condition is shown in Figure 3a,b, respectively.

Representative fluorescence images (20×) of an immobilized N2 worm recorded at 0 h, 15 h, and 30 h characterized the long-term evolution of the bacterial load and/or the evolution of the fluorescence signal due to the pH sensitivity of the fluorophore. At the beginning of the observation period (YA worm at *t* = 0 in Figure 3a,b), signals were very low along the whole intestinal lumen in both cases.

**Figure 4:**
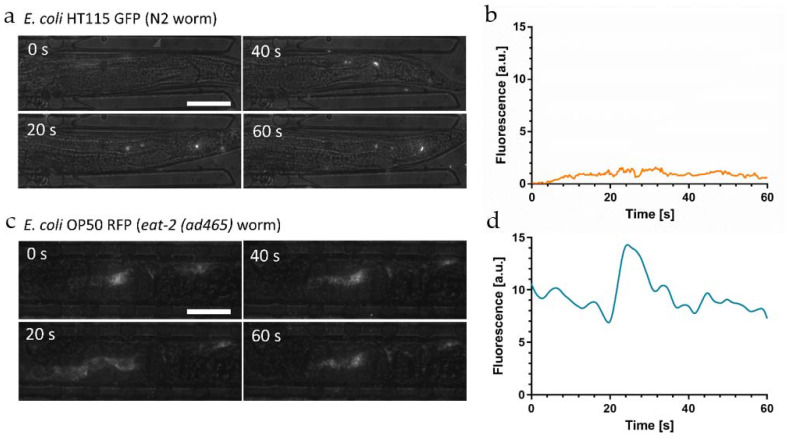
Intact vs. disrupted bacteria in the *C. elegans* intestine. Images and corresponding graphs are shown for a single representative worm for each condition. (**a**) High-resolution brightfield/fluorescence (50×) time-lapse images of an immobilized N2 *C. elegans* worm fed *E. coli* HT115 GFP. The image sequence shows a few intact bacteria passing through the hindgut region. Despite the pH sensitivity of GFP, fluorescence of intact bacteria is not affected by the acidic environment. Diffuse signals from disrupted bacteria that have lost membrane integrity cannot be observed due to fading of the GFP signal. (**b**) Fluorescent signal as a function of time measured in the worm in (**a**), showing a low but relatively constant GFP level originating from intact bacteria. (**c**) High-resolution time-lapse images of an immobilized *C. elegans* *eat-2* (*ad465*) mutant fed *E. coli* OP50 RFP. *eat-2* (*ad465*) mutants tend to accumulate intact bacteria in the posterior region of the intestine. The resulting fluorescent signal may be associated to a mixed load of intact and grinded bacteria, as the RFP marker is not affected by the low pH in the hindgut. (**d**) Time evolution of the fluorescent signal measured in the worm in (**c**). Overall, the fluorescence remains on a constant high level on a time scale relevant for food processing due to the stable presence of intact bacteria in the gut. The peak at *t*~25 s is due to ingestion and subsequent digestion of a new bacterial aliquot. Scale bars = 50 μm.

During the YA stage, worms are capable to disrupt and digest ingested bacteria efficiently without significant transient or permanent accumulation. Initial exposure conditions for RFP and GFP recording have been adjusted to the limit of detection of the bacterial load in the pharynx in either cases; thus, signal intensities of RFP and GFP raw images may be qualitatively compared. The pharyngeal pumping rate of adult *C. elegans* does not significantly differ for worms fed OP50 or HT115, respectively [22]. Thus, we assume that the amount of bacteria in a completely filled pharyngeal terminal bulb is comparable for both cases. During aging (15 h to 30 h in Figure 3a,b) a progressive increase in fluorescence was observed in the grinder and the hindgut regions for both cases, whereas the signal remained low in the mid-section of the intestine. The increase in fluorescence in the pharyngeal region may possibly be explained by the onset of a decreasing efficacy of the grinder function after 30 h. In fact, it was shown by analysis of incubated worm lysates that intestinal colonization levels, even for non-pathogenic *E. coli*, already increase at the early adulthood stage [21]. Moreover, pharyngeal swelling by bacterial infection has been identified as a reason for early death in *C. elegans* [52]. Furthermore, the hindgut region is particularly sensitive to bacterial proliferation fostered by senescent pathologies or genetic causes [21,28]. As will be discussed below, the strong fluorescent signal visible in the posterior region of the intestine of aging nematodes fed the OP50 RFP strain is generated by a mixture of fluorescent reporters originating from disrupted bacterial food and intact bacteria. Signals from both sources may persist in the hindgut due to the pH insensitivity of RFP. Color plot panels of the gut fluorescence corresponding to Figure 3a,b are shown in Figure 3c,d, respectively. These panels monitor the time evolution of the bacterial load more accurately with a time-lapse resolution of 30 min over 30 h. Overall, the signal intensity in nematodes fed OP50 RFP is considerably higher with respect to the HT115 GFP counterpart. Nevertheless, in both cases an onset of bacterial accumulation can be identified for *t* ≥ 20 h (Figure 3c,d). As the worm feeding behavior is not visibly impacted by the two *E. coli* strains, we conclude that the low GFP fluorescence in the hindgut is due to the pH sensitivity of this marker. In fact, only the diffuse GFP signal component related to membrane disruption is affected by the acidic environment (see discussion of Figure 4a); thus, we assume that the weak signal observed in this case is generated by a small amount of intact *E. coli* HT115 GFP persisting in the worm gut. The graphs shown in Figure 3e compare the evolution of the apparent average bacterial load over 30 h in worms fed *E. coli* OP50 RFP and HT115 GFP, respectively. The bacterial load increased over time in both cases, in particular for worms fed the OP50 RFP strain, reflecting mainly the strong increase in the hindgut signal. Overall, GFP values are below RFP values due to the pH sensitivity of the former. In Figure 3e, values have been normalized with respect to the maximum transient RFP or GFP fluorescent signal recorded in the pharynx, whereby we assume that these signals correspond to a comparable maximum amount of bacterial intake; therefore, both graphs in Figure 3e may be compared in a quasi-quantitative way.

We also recorded the bacterial load evolution in free-moving worms in the dedicated chambers on the chip. Figure S1b,c compares both conditions for the two *E. coli* strains and shows that worm immobilization did not affect food uptake behavior and bacterial load accumulation in the gut, indicating physiological conditions during immobilization. In order to verify whether the observed fluorescent signal in the gut reflects accurately the amount of ingested, disrupted, and digested bacteria, we investigated the impact of pH in more detail. In particular, as discussed above, for the pH-sensitive GFP reporter, the fluorescent signal may fade in the acidic worm gut, possibly falsely indicating a signal loss caused by chemical digestion. Here, we focus on single representative worms. Results obtained on larger populations showing bacterial distribution patterns in wild-type worms and *eat-2* mutants were presented in our previous paper [49]. Figure 4a shows a time-lapse sequence of high-resolution images (50×) of the hindgut of an immobilized YA N2 worm fed *E. coli* HT115 GFP.

In this image sequence, we could clearly resolve fluorescent spots generated by a few individual intact bacteria passing through the hindgut region, despite the high intestinal acidity in this region (pH~3.6). GFP fluorescence of intact bacteria thus appears not to be affected by the acidic environment. This observation indicated that the integrity of the bacterial membrane plays a significant role with respect to fluorescence emitted by the bacterial load during the ingestion and digestion process. On the other hand, no diffuse fluorescence associated to the presence of membrane fragments or other compounds originating from disrupted bacteria was observed in this case. GFP labeled bacteria thus turn out to be not suitable for monitoring digestion processes in the worm gut, as biochemical decomposition/nutrient absorption and fading of GFP signals due to low pH cannot be discriminated. The average fluorescent signal of the whole worm as a function of time is shown in Figure 4b, reflecting a low but relatively stable signal originating from intact bacteria persisting in the gut.

According to the findings presented above, digestion assays require worm feeding with the pH-insensitive OP50 RFP strain. In this case, we are able to observe strong fluorescent signals in the hindgut, caused by the presence of a mixture of disrupted bacteria and intact bacteria (Figure 3a). In order to confirm this hypothesis, we analyzed the bacterial load in *eat-2* mutants that are characterized by defective pharyngeal pumping, resulting in a conspicuous accumulation of live bacteria in the posterior region of the intestine [53]. Figure 4c shows a time-lapse sequence of high-resolution images (50×) of the hindgut of an immobilized *eat-2* mutant fed *E. coli* OP50 RFP.

We detected a mixed load of bacterial fragments and a high amount of intact bacteria generating superposed diffuse and clustered spot-like signals. The corresponding time evolution of the average fluorescent signal in the whole worm is shown in Figure 4d.

Overall fluorescence remains on a constant high level on a time scale relevant for food processing cycles due to the stable presence of a considerable number of intact bacteria in the gut of the *eat-2* mutant. The peak in Figure 4d is generated by a new food processing event, i.e., ingestion and subsequent digestion of a new bacterial aliquot.

### 4.2. Intestinal Load Dynamics and Biochemical Digestion of Bacterial Food

**Figure 5:**
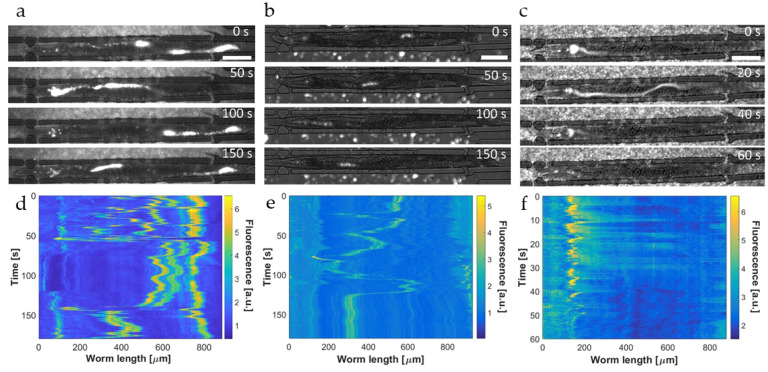
Analysis of the bacterial digestion and transit of indigestible microbeads in the worm intestine. (**a**–**c**) Representative fluorescence time-lapse low-resolution (20×) images of 3 YA N2 *C. elegans* worms immobilized in on-chip traps and fed *E. coli* OP50 RFP mixed with red fluorescent melamine microbeads (**a**), red fluorescent microbeads only (**b**), or *E. coli* OP50 RFP only (**c**), respectively. In (**b**) many beads located in the liquid chamber surrounding the worm can be seen. (**d**–**f**) Corresponding color plots of time-lapse recordings of the fluorescence intensity of the 3 nematodes in (**a**–**c**) showing the evolution and distribution of the bacterial load and/or the fluorescent beads. (**d**) Superposed diffuse and localized signals related to intact/disrupted bacteria and microbeads and (**e**) spatially constraint signal features generated only by microbeads. Microbeads are not digested in the gut and the periodic displacement of the intestinal load by peristaltic action, from the grinder to the hindgut and vice versa, can be tracked in both cases. (**f**) Fading of the fluorescent signal is observed after feeding the worm *E. coli* OP50 RFP only. Ingested bacteria initially accumulate in the pharynx and are subsequently pushed towards the posterior region of the gastrointestinal tract. A rapid signal decay occurs in the mid/posterior section of the intestine, starting at *t* > 30 s after the onset of peristalsis. This decay can be attributed to biochemical digestion, as the RFP label is not sensitive to the acidic environment in the gut. Scale bars = 100 μm.

**Figure 6:**
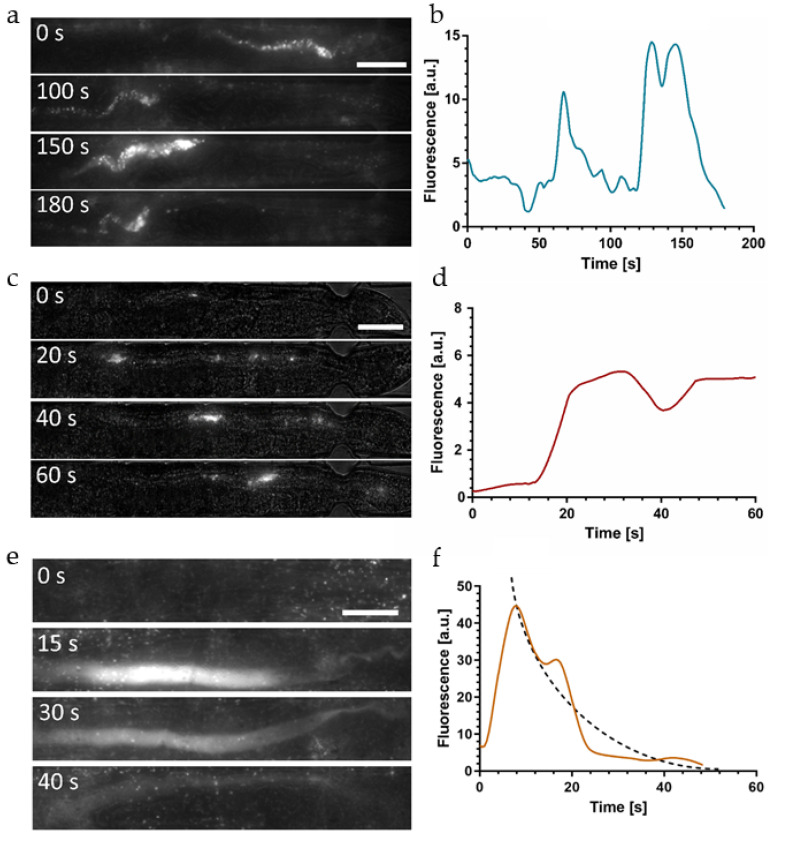
Analysis of transit patterns and bacterial nutrient absorption in the intestine of N2 *C. elegans* worms by high-resolution brightfield/fluorescence imaging (50×). Images and corresponding graphs are shown for a single representative worm for each condition. (**a**) Time-lapse images of the hindgut region of an immobilized YA worm fed a mixture of *E. coli* OP50 RFP and red fluorescent microbeads. A mixture of fluorescent microbeads and bacteria, residues of a previous ingestion, is visible at *t* = 0 s. Strong fluorescence observed at *t* = 150 s was caused by a subsequent peristaltic contraction, pushing intestinal load from the pharynx to the hindgut. (**b**) Average fluorescent signal in the hindgut region corresponding to (**a**). Peaks at *t* = 70 s and *t* = 130 s are caused by transient microbead/intact bacteria clusters (initially located in the proximity of the rectal region, at *t* = 0 s in this image sequence), which were repeatedly pushed back to the pharynx by peristaltic activity. (**c**) Images of the hindgut of a YA worm fed indigestible microbeads only, showing transfer of the beads to the posterior gut region shortly after ingestion. (**d**) Average fluorescence signal of the hindgut region corresponding to (**c**). The intensity remained stable over this short initial time period (60 s) after indigestion, since further peristaltic events did not yet occur. (**e**) Time-lapse images of the hindgut region of an immobilized YA worm fed *E. coli* OP50 RFP only. An aliquot of disrupted bacteria appearing in the posterior gut section generates strong diffuse fluorescence (*t* = 15 s). A gradual decay of fluorescence over time was observed (*t* = 30 s and 40 s), associated to biochemical digestion of membrane fragments and nutritive compounds. (**f**) Fluorescent signal corresponding to (**e**) recorded in the hindgut region. Based on additional data (Figure S7, *n* = 5), an exponential decay curve (dashed line) was defined in order to determine the time constant for food digestion (*τ* = 14 ± 4 s). Scale bars = 50 μm.

Based on the imaging protocols and preliminary studies presented above, we were able to evaluate the time constant of nutrient absorption in the worm intestine. We designed an assay for observing simultaneously the fate of *E. coli* OP50 RFP fluorescence due to enzymatic digestion and the intestinal peristaltic action. For the latter, indigestible 1 µm-sized rhodamine B-marked melamine microbeads were used for tracking the dynamics and cyclic motion of the intestinal load. In order to obtain real-time recordings of the digestive processes, we performed short time-lapse imaging sequences (60 s to 180 s, frame interval of 250 ms) of confined YA N2 *C. elegans* worms, either at low resolution (20×) for a global view of the entire worm intestine (Figure 5), or at high resolution (50×) for local and more precise observations, and in particular for the measurement of the decay of the diffuse fluorescent signal in the hindgut (Figure 6). After worm loading and trapping, on-chip microfluidic units were filled either with (i) a mixture of *E. coli* OP50 RFP and red fluorescent microparticles (~4 × 10^9^ beads/mL in S-medium) in equal proportions, (ii) fluorescent microparticles only, or (iii) *E. coli* OP50 RFP only, respectively. The chip was refilled with fresh suspension every 10 min in order to maintain the amount of bacteria or particles in the microfluidic chambers.

Representative low-resolution fluorescence images for all three conditions are shown in Figure 5a–c, respectively. Figure 5d–f are the corresponding color plots. When mixed, intact bacteria and microbeads cannot be distinguished in the worm intestine. In this case, the recorded signal sequences also show extended diffuse regions originating from bacterial membrane fragments. Fluorescent spots and diffuse signals are superposed, resulting in relatively broad features in the intestine (Figure 5a,d). One the other hand, in the case of mere microbeads ingestion, spatially constrained features with stable fluorescence intensity were observed and bright spots generated by single beads and bead clusters can be more clearly identified in the absence of a diffuse signal (Figure 5b,e). In both cases, resin microbeads cannot be digested and are periodically displaced by peristaltic contractions from the pharyngeal region to the hindgut and vice versa (Figure 5d,e, see also Figure 6b). Color panels similar to Figure 5d were used to evaluate the periodicity of the intestinal load motion, and for that, the time evolution of the signal average over the hindgut region was plotted. Four representative graphs are reported in Figure S5, from which we determined an average period of *T*_p_ = 71 ± 5 s. In Supplementary Video S1 multiple peristaltic contractions in the posterior intestinal lumen of a single N2 YA *C. elegans* worm were recorded. Such periodical muscular contractions generate the observed cyclic motion of the intestinal load through the entire worm gut. On the contrary, when worms are fed only *E. coli* OP50 RFP bacteria, the transient pattern of the observed fluorescent signals is very different and determined by the rapid biochemical digestion of bacterial food. This dynamic and kinetic behavior is exemplified in the image sequence shown in Figure 5c and with higher time resolution in the color plot panel of Figure 5f. Ingested bacteria, initially intact and concentrated in the worm pharynx, are physically disrupted by mechanical action of the grinder and subsequently, after passing the pharyngeal-intestinal valve, pushed towards the posterior region of the gastrointestinal tract until the pharyngeal lumen is empty. Strongest bacterial transfer from the pharynx to the adjacent intestinal lumen is visible in the time span between 10 s and 30 s on the color plot panel of Figure 5f. This event was also captured in the image at 20 s in Figure 5c.

Subsequently, a rapid decay of the signal corresponding to the transferred amount of bacteria was observed. Fluorescent signal fading can be mainly identified in the gut section extending from the midgut to the rectum, where already 45 s after a food ingestion event and the onset of peristalsis (*t* = 0 in the image sequence), the intestine is almost free of fluorescence (Figure 5c,f for *t* ≥ 45 s). The bacteria-associated fluorescent signal becomes negligible after 60 s. Because of the insensitivity of the RFP marker to the relatively strong acidic pH in the mid and posterior region of the intestine, the observed signal decay has to be attributed to the process of nutrient metabolization and absorption, i.e., the activity of digestive enzymes that decompose fluorescent bacterial membrane fragments and nutritive compounds released from the cytoplasm. Defecation did not take place on the time scale of this recording. For these assays, the examined worms were in the YA stage where notable fluorophore/bacterial accumulation in the hindgut did not occur (as was observed for somewhat older worms in Figure 3a,c). As mentioned above, diffusive fluorescent features can also be identified for the mixed intestinal load monitored in Figure 5d. In principle, it should also be possible to detect a decay of fluorescence in this color plot panel. However, relatively strong bead signals impede to a certain extent accurate observation of weaker signal variations due to digestion. Furthermore, recording was carried out over a longer time scale in this case, involving successive ingestion events that replenish the bacterial load in the intestine and cyclic peristaltic motion, eventually screening somewhat a digestive decay.

A more detailed investigation of the bacterial transport and digestion processes addressed in Figure 5 is presented in Figure 6 based on high-resolution (50×) time-lapse imaging of the hindgut region of immobilized YA worms. As before, the worms were fed a mixture of *E. coli* OP50 RFP and red fluorescent melamine microbeads (Figure 6a), fluorescent microbeads only (Figure 6b), or *E. coli* OP50 RFP only (Figure 6c). In the mixed state shown in Figure 6a, single dots and fluorescent clusters, generated by resin microparticles and a small amount of intact bacteria, and diffuse fluorescence associated with grinded bacteria are overlapping and can hardly be discriminated. In this case, the time sequence has been recorded over 180 s in order to focus on the periodicity of motion in the gut due to peristaltic action. The corresponding time evolution of the fluorescent signal in the hindgut region is shown in Figure 6b. The peak at *t* ≈ 70 s and the region extending from *t* ≈ 120–160 s (also captured in the image at 150 s in Figure 6a for instance) are caused by the transient localization of clusters of fluorescent microbeads/intact bacteria in the hindgut that are repeatedly pushed back towards the pharynx.

The somewhat asymmetric shape of the peaks, due to a slower decay of the peaks with respect to the peak rise, might possibly be related to a digestive process superposing the peristaltic activity (see discussion below and Figure 6f). The cyclic motion of the internal load through the entire worm gut could also be clearly seen in the color plots of Figure 5d,e. Considering the time scale of these recordings (180 s), it appears that not-grinded intact bacteria and indigestible beads somehow hinder the defecation process, which normally occurs on a time scale of about 50 s [17].

A closer view on the initial phase after food ingestion is shown in the time-lapse sequence of Figure 6c (50×, 60 s). In this case, the immobilized YA N2 worm was fed red fluorescent microbeads only for visualizing the dynamic behavior of the initial phase after food uptake where beads have been transported to the posterior gut section after passing the grinder without damage. Since digestion of beads does not occur, the fluorescence intensity averaged over the hindgut region is expected to be stable.

Figure 6d shows that the time evolution of fluorescence approximates a step-like curve, corresponding to the appearance of the first bead cluster in the hindgut (at *t* ≈ 20 s in this case) and the temporary stability of the signal (*t* ≈ 20–60 s). Subsequently, microbeads are pushed back toward the grinder by a peristaltic contraction, corresponding to the situation shown in Figure 6a,b. Defecation may also happen but was not observed in this case. On the other hand, additional transitory accumulation in the posterior intestine could be observed if another consecutive ingestion event occurs rapidly. As shown in two of the graphs in Figure S6, the fluorescence curves feature a two-step shape in the latter case.

In order to accurately quantify the time constant *τ* associated with the digestive process of enzymatic and biochemical transformation of disrupted bacterial food, we acquired high-resolution images (50×) of the hindgut area of immobilized YA N2 worms fed with *E. coli* OP50 RFP only. Figure 6e shows a representative image sequence visualizing the evolution of the fluorescent signal. The focal plane was adjusted accurately to the mid-height of the gut in order to correctly capture the possible presence of intact bacteria.

The diffuse fluorescence signal was extracted from the raw images according to the image processing protocol described in Figure 2c–f. At *t* = 0 food indigestion occurred and no bacteria-related signal could be observed in the gut at this stage. At *t* = 15 s strong diffuse fluorescence filled the whole intestinal lumen in the posterior region. As was discussed earlier, we assume that this diffuse signal is generated by a mix of small membrane fragments of disrupted bacteria, a significant amount of partly dissociated membrane compounds that still incorporate active RFP markers, and possibly fluorescent cytoplasmic content that was released after disintegration of the bacteria.

Subsequent images (Figure 6e, *t* = 30 s and 40 s) revealed a rapid decay of the fluorescent signal in the intestine. We attributed the fading of intensity to chemical digestion and absorption of nutrients in the worm gut.

Other possible reasons for a loss of signal, in particular pH sensitivity or photobleaching of RFP, relocation of the intestinal load outside the field of view due to peristaltic action (appears only on a longer time scale), and defecation (not observed before the complete fluorescence clearing in this case) can be excluded. In Figure 6f, we quantitively analyzed the time dependence of the diffuse fluorescent signal intensity averaged over the hindgut, corresponding to the image sequence in Figure 6e. The initial peak at *t* = 15 s indicates the arrival of the bacterial load in the hindgut after food uptake and grinding. In the time span of 15 s to 50 s the plot shows the rapid signal decay, which becomes negligible for *t* > 50 s in this case. The exact features of such graphs are subjected to natural variations of the biological processes and a lack of precision of certain assay parameters (e.g., defining the exact time point of food uptake or the transient bacterial accumulation phase in the grinder). Additional typical examples for different worms are reported in Figure S7. All graphs are clearly different from the step-like shape observed for indigestible beads reflecting a constant fluorescent signal after ingestion (Figure 6d). By averaging the recorded data, we defined an exponentially decaying curve (dashed line in Figure 6f) and estimated a time constant *τ* = 14 ± 4 s (*n* = 5) for biochemical food digestion in the *C. elegans* intestine. This result may also be qualitatively compared with Figure 4d for *eat-2* pharyngeal-pumping defective mutants. In the latter case, the stable baseline of the fluorescent signal, reflecting temporary accumulation of intact bacteria in the hindgut, was superposed by a transient peak due to a new aliquot of grinded bacteria that were rapidly digested.

### 4.3. Infection Assay Based on M. marinum Accumulation and hsp-6::gfp Expression

A wide variety of bacterial species may infect *C. elegans,* which has therefore evolved to an important model for studies of host immunity mechanisms and of pathogen virulence that are analogous to those involved during pathogenesis in humans [23,24,25]. In order to validate our microfluidic platform and experimental methods with respect to specific applications, in particular for infection assays involving studies of pathogenic intestine colonization and for monitoring pathogen-induced mitochondrial stress in *C. elegans*, we designed a series of experiments based on the important aquatic pathogen *M. marinum. M. marinum* is a slow-growing non-tuberculous mycobacterium that causes a tuberculosis-like illness in fish [54]. In particular, the zebrafish is a natural host to *M. marinum* that was used as a model to study tuberculosis pathogenesis, as well as for antitubercular drug discovery [55]. In humans, *M. marinum* is medically important as it causes infections ranging from cutaneous granulomatous lesions to debilitating disseminated infections, possibly progressing to more invasive diseases such as tenosynovitis, septic arthritis, or osteomyelitis [56]. *M. marinum* is also pathogenic to *C. elegans*. For instance, it was shown that *C. elegans* infected with *M. marinum* displayed a highly increased mortality rate within two days post-infection [57]. Our assays consist of feeding on-chip immobilized worms with the pathogen and measuring (i) the evolution of bacterial accumulation in the worm intestine over several hours, and (ii) quantifying specific fluorescent phenotypic worm features by imaging the time evolution of *hsp-6::gfp* expression, which is an important reporter of mitochondrial stress and the activation of the mitochondrial unfolded protein response (UPR^mt^) in *C. elegans* [34].

For the colonization assays, approximately 16 N2 worms were loaded in each assay unit of the device. Two units were filled with GFP labeled *M. marinum* suspended in S-medium. Considering our discussion above, the pH sensitivity of the GFP fluorophore related to disrupted bacteria is not an issue in the present study, as only accumulation of intact live bacteria is of interest, which are not affected by the acidic environment of the intestine. Non-fluorescent *E. coli* OP50 was used as reference for normal feeding conditions of the worm control groups in the other two on-chip assay units. We acquired high-resolution (50×) fluorescence images of the gut over 15 h, specifically of the pharyngeal and posterior regions, since these sections are most sensitive to bacteria accumulation.

**Figure 7:**
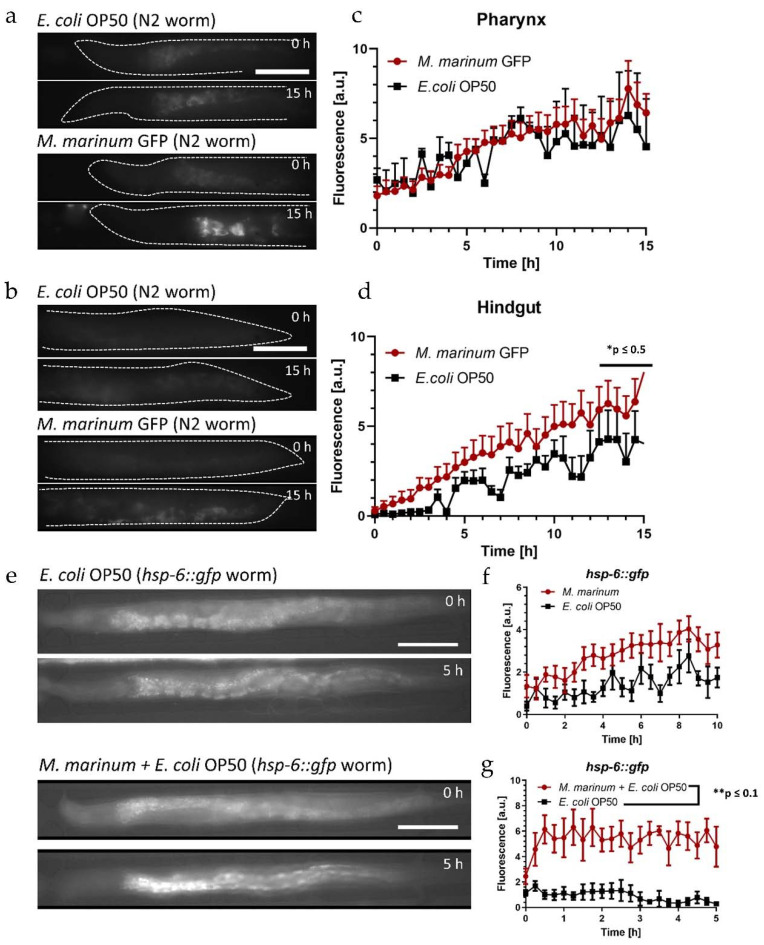
Analysis of bacterial accumulation and UPR^mt^ response in *C. elegans* nematodes induced by pathogenic *M. marinum* bacteria. (**a**,**b**) Fluorescence images (50×) of pharynx (**a**) and hindgut (**b**) of an immobilized YA N2 *C. elegans*, fed OP50 *E. coli* bacteria or GFP expressing *M. marinum*, respectively. Worm contours are indicated by dashed lines. Accumulation of *M. marinum* in the grinder and in the hindgut can be identified after 15 h. (**c**,**d**) Time evolution of the bacterial load in the pharynx (**c**) and in the hindgut (**d**). Average fluorescent signals were normalized with respect to the *E. coli* OP50 value measured in the pharynx for each time point (* *p* ≤ 0.5, *n* = 10). (**e**) Fluorescence images of immobilized YA *hsp-6::gfp* mutants fed *E. coli* OP50 only (top images) and a mixture of *M. marinum* and *E. coli* OP50 (bottom images). The immobilized worms are oriented with head to the left. Strong fluorescence due to *hsp-6::gfp* expression in the worm fed *M. marinum* is visible in the anterior region of the intestine adjacent to the pharynx. (**f**) Average fluorescence signal related to *hsp-6::gfp* expression for nematodes fed *M. marinum* only and *E. coli* OP50 only, respectively (*n* = 5). (**g**) Fluorescence signal related to *hsp-6::gfp* expression for nematodes fed a mixture of *M. marinum*/*E. coli* OP50 and with *E. coli* OP50 only, respectively. *E. coli* OP50 were added to *M. marinum* to verify that the increase in *hsp-6::gfp* expression is not caused by worm starvation (** *p* ≤ 0.1, *n* = 5). Error bars correspond to mean ± SEM. Scale bars = 100 μm.

Images for both gut sections immediately after food uptake (*t* = 0 h) and after on-chip incubation (*t* = 15 h) are shown in Figure 7a,b, respectively. The evolution of the weak autofluorescent signal in a worm fed *E. coli* is hardly visible with exposure conditions optimized for *M. marinum* (Figure 7a, upper panel). On the other hand, bright spots due to localized accumulation of intact *M. marinum* GFP in the grinder can be seen after 15 h (Figure 7a, lower panel). Likewise, the *M. marinum* bacteria load increased in the hindgut, but the signal is distributed over a larger portion of the intestine, and thus is relatively weak (Figure 7b, lower panel, same exposure conditions as in Figure 7a). The graphs in Figure 7c,d express more clearly the time evolution of the corresponding signal intensities, averaged over the pharyngeal and posterior regions, respectively. The *E. coli* autofluorescence control signal increased over time indicating a progressive accumulation of bacteria in the grinder (Figure 7c, black curve). The observation of this phenomenon was more evident in Figure 3b (at *t* = 30 h, using *E. coli* HT155 GFP). The evolution of the fluorescent signal associated with *M. marinum* in the pharynx (Figure 7c, red curve) was similar to *E. coli*. For a better comparison, *M. marinum* values were normalized for each time point with respect to the *E. coli* OP50 intensity determined in the grinder. Therefore, we deduce from Figure 7c that no specific additional accumulation occurred in the grinder for the present assay conditions. On the other hand, the normalized *M. marinum* signal in the hindgut increased faster than the *E. coli* control (Figure 7d), indicating accumulation and/or colonization possibly due to the pathogenic effect of *M. marinum*. In both cases, a rise of the fluorescent signal in the worms could in principle also be partly be due to increasing autofluorescence in aging worms, however, at the early adulthood stage of the worms in the present assays this effect should be negligible [58].

In order to evaluate the feasibility of recording UPR^mt^ response with our microfluidic platform, we first measured the mitochondrial stress response in YA *hsp-6::gfp* mutants caused by prolonged exposure to tetracycline. For the assay, an *E. coli* suspension was mixed with tetracycline to a final concentration of 60 μg/mL. Corresponding brightfield/fluorescence images are shown in Figure S8, where a bright signal generated by *hsp-6::gfp* expression in the pharyngeal region could be observed after tetracycline exposure for 8 h (Figure S8a, lower panel). The increase in *hsp-6::gfp* expression of the treated worms over 15 h is represented in the graph of Figure S8b. To evaluate the mitochondrial stress response induced by pathogenic *M. marinum*, we monitored the fluorescent *hsp-6::gfp* signal in YA mutants for different feeding conditions. Fluorescence images of immobilized *hsp-6::gfp* mutants acquired after the initial filling of the chip with different bacterial suspensions (*t* = 0 h) and after incubation for 5 h are shown in Figure 7e. Images of a *hsp-6::gfp* mutant fed *E. coli* OP50 only did not show a significant difference over the time interval of 5 h (Figure 7e, top panel). The time evolution of fluorescence intensities for *hsp-6::gfp* mutants fed *M. marinum* only and the *E. coli* control group is represented in the graph of Figure 7f (averaged over the entire worm). Overall, the induced fluorescence response was higher with *M. marinum* compared with *E. coli* only, showing a slight increase over time, similar to the effect of tetracycline (Figure S8b). In order to verify that mitochondrial stress in worms was not induced by food avoidance and starvation, the infection assay was repeated with a mixed suspension of *M. marinum* + *E. coli* OP50 thus delivering the pathogen and providing an adequate food source simultaneously. Fluorescence images of an immobilized *hsp-6::gfp* mutant are shown in Figure 7e (bottom panel). Strong fluorescence due to *hsp-6::gfp* expression in the anterior region of the intestinal track is clearly visible after 5 h. The corresponding fluorescence signal averaged over the whole worm is shown in the graph of Figure 7g (red curve). Similar to *M. marinum* only (Figure 7f), the *hsp-6::gfp* induced signal was above the control values for feeding with *E. coli* only. However, in the latter case, we observed a faster onset of stress-related *hsp-6::gfp* expression and a higher overall fluorescent intensity. This could possibly be due to a higher uptake of *E. coli* being a suitable food source, entailing simultaneous ingestion of an enhanced amount of *M. marinum* in the mixed feeding suspension, and thus resulting in an enhanced stress response.

## 5. Discussion

Developing new versatile tools for investigating in vivo the bacterial load in the *C. elegans* intestinal track appears to be important in the present context, where *C. elegans* is increasingly explored as a model organism for the microbiota–gut interaction and for infections assays related to human pathogenesis. Aside from assessing typical worm phenotypes, development and health indicators, imaging bacterial populations, and properties directly inside the gut under different pharmacological or microbiological conditions provide important new information. Digestion or food absorption parameters, detecting small amounts of persisting live bacteria, or gut colonization by pathogens are relevant features in this perspective.

Conventional tools and assays, mainly based on bacteria-seeded NGM plates, have been used for the observation and measurement of bacterial colonies in the worm intestine [8,9,10]. However, for the time being, a very limited number of related studies have been performed by taking advantage of microfluidic approaches, despite the fact that microfluidic tools have been successfully used in *C. elegans* research for a large variety of topics, ranging from behavioral to neurobiological studies [59,60]. For instance, in microfluidic antimicrobial assays, only representative low-resolution images of the overall bacterial load in infected *C. elegans* have been shown, possibly taken off-chip without worm immobilization [45,46]. Lee et al. developed a hexagonal array of micropillars that supported worm motility. The post-infection *P. aeruginosa* bacterial load in the intestine was displayed at low resolution [43]. In another infection assay based on the WormSpa, worms were immobilized and representative low-resolution brightfield images of a single worm fed *P. aeruginosa* were presented [44]. To our knowledge, the work presented here is the first study that analyzes the bacterial load dynamics, digestion, and food absorption in the *C. elegans* gut directly by means of a microfluidic device. Our approach allowed both long-term low-resolution time-lapse imaging of the entire worm body and high-resolution imaging of small sections of the worm intestine for several hours.

We identified and characterized dynamic features, in particular cyclic motion of bacteria clusters in the gut due to peristalsis. Detection of single live bacteria, discrimination between intact bacteria, and disrupted bacterial fragments in the posterior intestinal lumen, bacterial absorption, and bacterial accumulation in different sections of the worm were successfully monitored. Worm response to pathogenic bacteria was also analyzed.

A major challenge of such on-chip assays is performing in vivo long-term immobilization of *C. elegans* nematodes, while maintaining the worms in healthy and physiological conditions. Continuous nutrition supply and frequent egg laying are concomitant factors that are specifically relevant for long-term on-chip culture and observation. Moreover, starting from the YA stage, *C. elegans* are able to exert significantly higher force with respect to the larval stages [61], especially with their mid-body and tail regions, which further complicates stable immobilization of the worms over extended periods. The microfluidic device presented in our work proved to be suitable for in vivo bacterial imaging and for measuring the bacterial load in the *C. elegans* gut in a dynamic way. As no active elements are involved for immobilization, device fabrication and operation were simple. Continuous feeding during worm trapping was not impeded. We took advantage of specific features of a microfluidic trap design presented earlier by Kopito et al. [41]. In particular, perforating the sidewalls of the traps allowed continuous egg laying, an important requirement for enabling physiological conditions during worm confinement. We adapted and optimized our trap design by introducing dedicated passive structures (trapping pillars) at the entrance of each worm trap, enabling secured worm confinement without applying continuous flow thus making fluidic protocols more robust. As the worm is intentionally not tightly immobilized or compressed, a slight margin of worm movement is allowed during on-chip confinement, e.g., small rotations or longitudinal displacements along the trapping channel. Certain limitations might be encountered for tracking of non-fluorescent or not easily identifiable anatomical worm features, however, for the present application such issues were not relevant. In any case, the intrinsic limit for high-resolution imaging of the worm gut is set by the internal natural residual motion of the organs and, more importantly, the dynamic behavior of the bacterial load to be studied. Moreover, additional culture chambers have been implemented on the chip, enabling simultaneous measurements of conventional phenotypes related to free-moving worms, e.g., thrashing frequency or motility, under identical feeding conditions as for confined worms. Such an approach obviates more complicated reversible immobilization protocols.

Implementing microfluidic approaches for advanced and accurate in vivo imaging of bacterial colony dynamics and digestion kinetics opens the path to enhance the information content, throughput, and versatility of assays targeting *C. elegans* microbiota–gut interaction. For instance, microfluidic platforms would be especially suitable for parallel on-chip assays based on worm feeding with different bacterial strains and/or the application of different antimicrobial compounds [62]. Likewise, a range of *C. elegans* mutants, presenting specific drug-dependent microbial–host interaction features, could be observed simultaneously.

## 6. Conclusions

We proposed a versatile method for the analysis of intestinal processing of bacterial food in *C. elegans* nematodes. Our microfluidic platform enabled both imaging and tracking of the dynamics of the bacterial load in the entire worm gut and local high-resolution observations in specific sections, in particular in the pharynx and the hindgut. The time evolutions of fluorescence patterns were measured in worms fed *E. coli* bacteria marked either with pH-insensitive (RFP) or pH-sensitive (GFP) reporters, respectively, in order to determine reliable assay protocol conditions. Ingestion of mixed suspensions of bacteria and indigestible microbeads enabled monitoring dynamic features related to the peristaltic activity of the worm gut. Our imaging protocol allowed discriminating between intact *E. coli* bacteria and diffuse fluorescent signals due to bacterial food disrupted by grinding. By recording the decay of the diffuse signal intensity, we could estimate the time constant for enzymatic digestion in N2 YA *C. elegans,* which was of the order of 14 s. As a further validation of our protocols, and in view of possible applications for infection assays, we investigated the bacterial load in the worm intestine in the presence of pathogenic *M. marinum* bacteria and measured the expression of mitochondrial stress response in *C. elegans hsp-6::gfp* mutants. We envision that our system provides significant added value for advanced bacteria-based *C. elegans* assays focusing on time-lapse imaging of specific microbia–gut interactions, nutrient absorption kinetics, and the dynamics of the bacterial load in the gut. For instance, such features may be used for assessing the virulence of different pathogenic strains in the *C. elegans* model organism or for the evaluation of antimicrobial drug efficiency in vivo.

## Supplementary Materials

The following are available online at https://www.mdpi.com/article/10.3390/mi12070832/s1, Figure S1: Monitoring of physiological parameters of trapped and free-moving N2 worms, Figure S2: Comparison between two different ways of light exposure, Figure S3: Comparison between *E. coli* OP50 RFP fluorescence and C. elegans autofluorescence, Figure S4: Discrimination of intact and disrupted *E. coli* OP50 RFP bacteria, Figure S5: Evaluation of the periodicity of the intestinal load displacement due to peristaltic activity, Figure S6: Worms fed with fluorescent microbeads, Figure S7: Worms fed with *E. coli* OP50 RFP, Figure S8: UPRmt response in *C. elegans hsp-6::gfp* mutants induced by the antibiotic tetracycline, Video S1: Peristaltic intestinal contractions of a *C. elegans* worm.
