# Supplementary material for: An In Vivo Microfluidic Study of Bacterial Load Dynamics and Absorption in the C. elegans Intestine
Source: Micromachines (Basel). 2021 Jul 17;12(7):832. doi: 10.3390/mi12070832 (PMC8304684; doi:10.3390/mi12070832)
Supplement: Supplementary file 1 [file micromachines-12-00832-s001.zip › micromachines-1293576-supplementary/supplementary_figures_pr.pdf]

Supplementary Information

An In Vivo Microfluidic Study of Bacterial Load Dynamics and Absorption in the *C. elegans* Intestine

Vittorio Viri, Maël Arveiler, Thomas Lehnert, Martin A. M. Gijs*

Laboratory of Microsystems, Ecole Polytechnique Fédérale de Lausanne,
CH-1015 Lausanne, Switzerland.

*Author to whom correspondence should be addressed

E-mail: martin.gijs@epfl.ch

Supplementary Video S1. Peristaltic intestinal contractions of a *C. elegans* worm. Brightfield video recording of the posterior worm section of a gravid N2 worm recorded over 60 s, showing multiple peristaltic contractions of the intestinal lumen. Such periodic muscular contractions determine the dynamics of the intestinal transport and may generate, for instance, cyclic motion of the intestinal load through the entire worm gut (as monitored in Figure 5d and 5e). An initial contraction of the hindgut is visible at $t \approx 10$ s, and a second one occurs at $t \approx 60$ s.

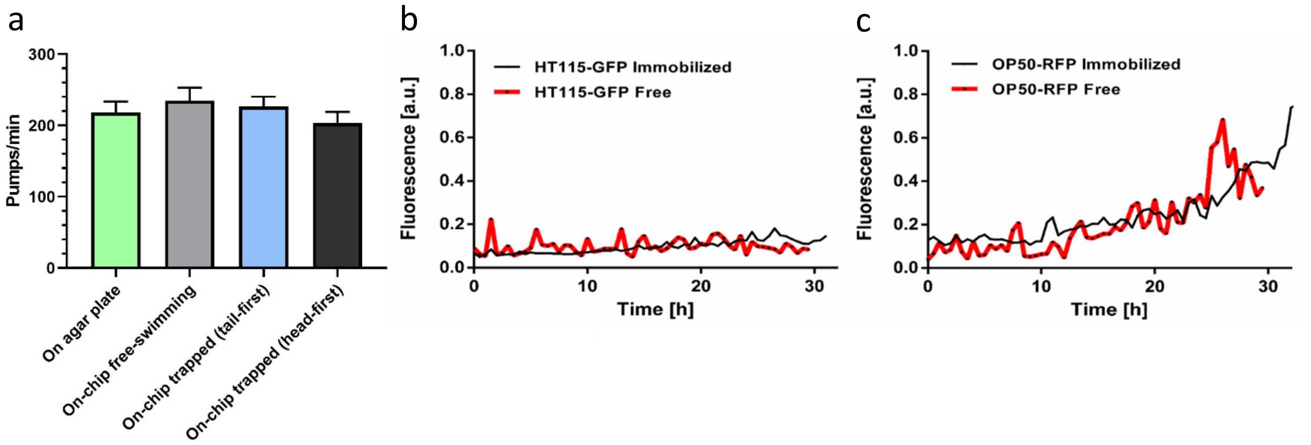

Figure S1. Monitoring of physiological parameters of trapped and free-moving N2 worms. (**a**) Comparison between the pharyngeal pumping rates measured in YA N2 fed with *E. coli* OP50 RFP cultured on agar plate and on-chip. Measurements on the chip were performed on worms cultured in the free-worm chambers and on worms confined in the trapping channels (for head-first and tail-first body orientations), respectively. Head-first orientation is shown in Figure 1d. Measurements on-chip were acquired 1 hour after worms loading. Error bars correspond to mean ± SD, $n = 10$. (**b**,**c**) Comparison between the bacterial load measured by fluorescence of the gut of immobilized and free-swimming worms in adult N2 fed with *E. coli* HT115 GFP (**b**) and *E. coli* OP50 RFP (**c**) over 30 h. Fluorescence values were averaged over the whole intestine and normalized with respect to the maximum fluorescence value, measured in the pharynx. Immobilization is not significantly affecting eating and bacterial load in the worm gut in both cases.

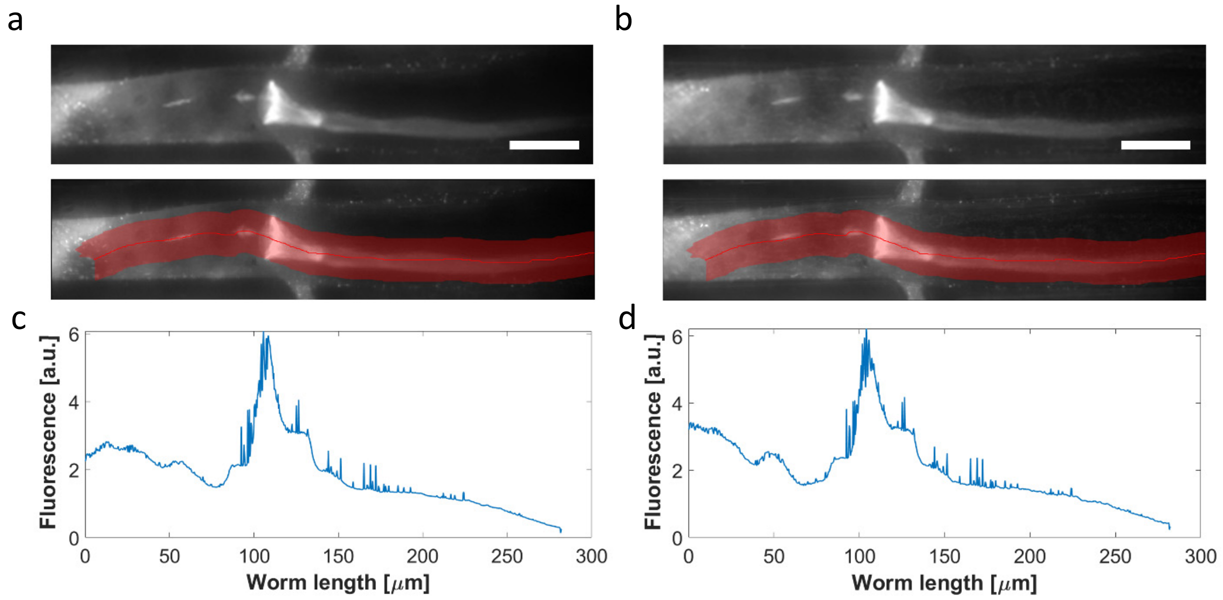

Figure S2. Comparison between two different ways of light exposure. Fluorescent images of the pharyngeal lumen of an N2 adult worm filled with RFP expressing OP50 *E. coli* bacteria, recorded with double-light exposure (white and 545 nm green excitation light) (**a**), and with 545 nm green light excitation only (**b**). The fluorescent signal was measured along the 25 µm-wide red line (bottom parts of a, b). (**c**,**d**) Comparison between fluorescent signals measured along the red line with double-light exposure (**c**), and with fluorescence excitation only (**d**). By keeping the power of the white light source low, the difference between the two signals was negligible. Scale bar = 40 µm.

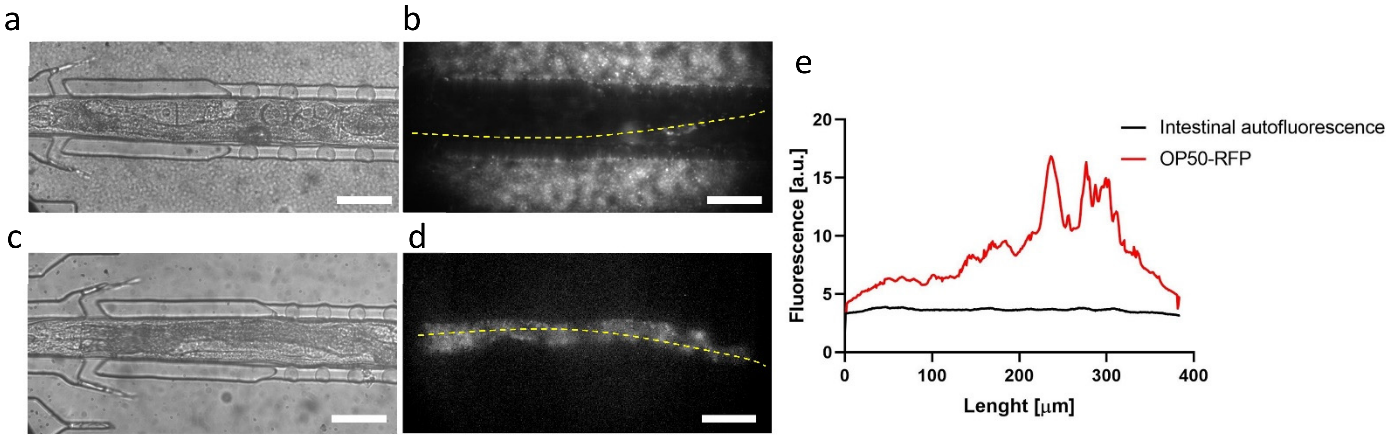

Figure S3. Comparison between *E. coli* OP50 RFP fluorescence and *C. elegans* autofluorescence. Brightfield and fluorescent images of the pharyngeal lumen and midgut of a N2 YA worm filled with RFP expressing OP50 *E. coli* bacteria (**a**,**b**), and of a N2 YA worm in buffer solution displaying intestinal autofluorescence (**c**,**d**). The contrast of the image in (**d**) was enhanced with respect to (**b**) in order to make intestinal autofluorescent lipid droplets visible. Scale bars = 50 µm. (**e**) Comparison between fluorescent signals generated by the presence of bacteria (**b**) and intestinal autofluorescence (**d**) measured in the worm bodies along the dashed lines.

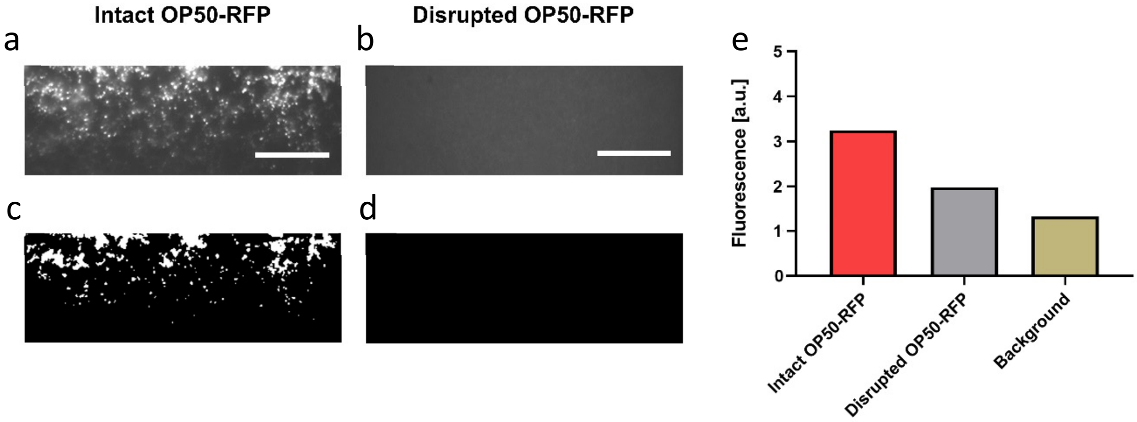

Figure S4. Discrimination of intact and disrupted *E. coli* OP50 RFP bacteria. Fluorescent images of intact RFP expressing OP50 *E. coli* bacteria suspended in buffer solution (**a**), and of bacteria disrupted by exposure to Virkon® 1% solution (**b**). Virkon® disrupts the bacterial cell membrane. No distinct fluorescent spots can be distinguished in the latter case. Scale bar = 40 µm. (**c**,**d**) Images obtained after application of the brightness level thresholding protocol to intact (**c**) and disrupted (**d**) OP50-RFP *E. coli* bacteria. The higher fluorescent signal generated by intact bacteria allows clear detection of single bacteria or agglomerates of fluorescent bacteria and background removal. (**e**) Comparison between average fluorescent values measured in (**a**), (**b**) and background measured in the absence of bacteria.

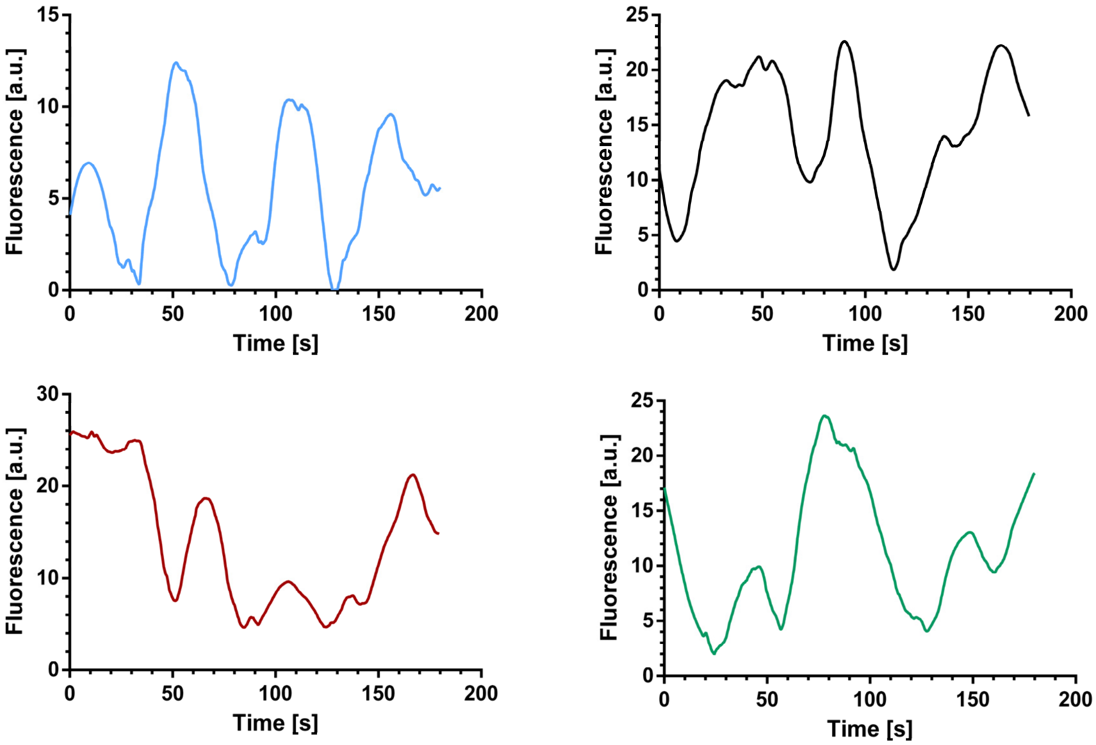

Figure S5. Evaluation of the periodicity of the intestinal load displacement due to peristaltic activity. Color panels like shown in Figure 5d were used to evaluate the time evolution of the average fluorescent signal corresponding to the hindgut region. Results for 4 representative worms are shown, fed with a mixed suspension of RFP expressing *E. coli* OP50 bacteria and red fluorescent rhodamine B-marked melamine microbeads. We determined an average period of $T_P = 71 \pm 5$ s.

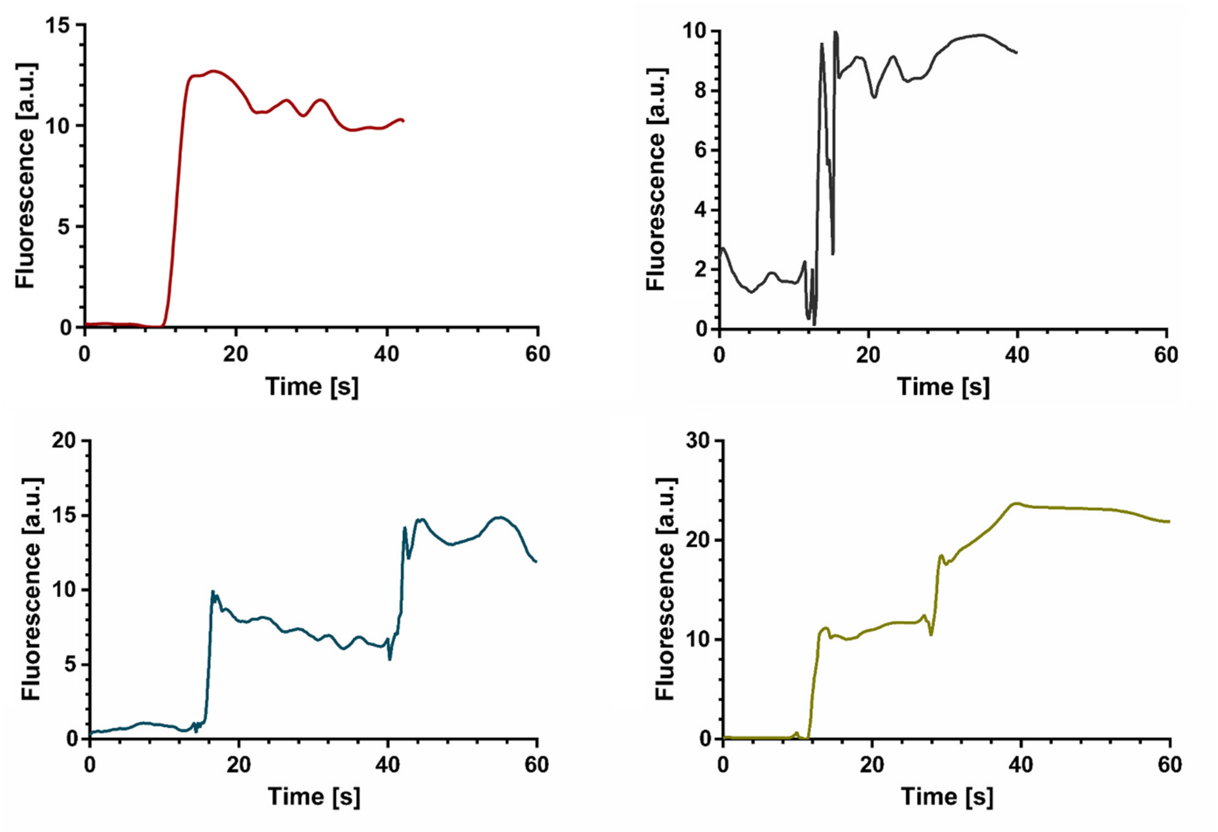

Figure S6. Worms fed with fluorescent microbeads. Fluorescent signal corresponding to the hindgut region for 4 representative worms. In the two lower graphs, the 2-step shape of the fluorescent signal increase is caused by two consecutive ingestion events of indigestible beads (spaced by ~ 20 s).

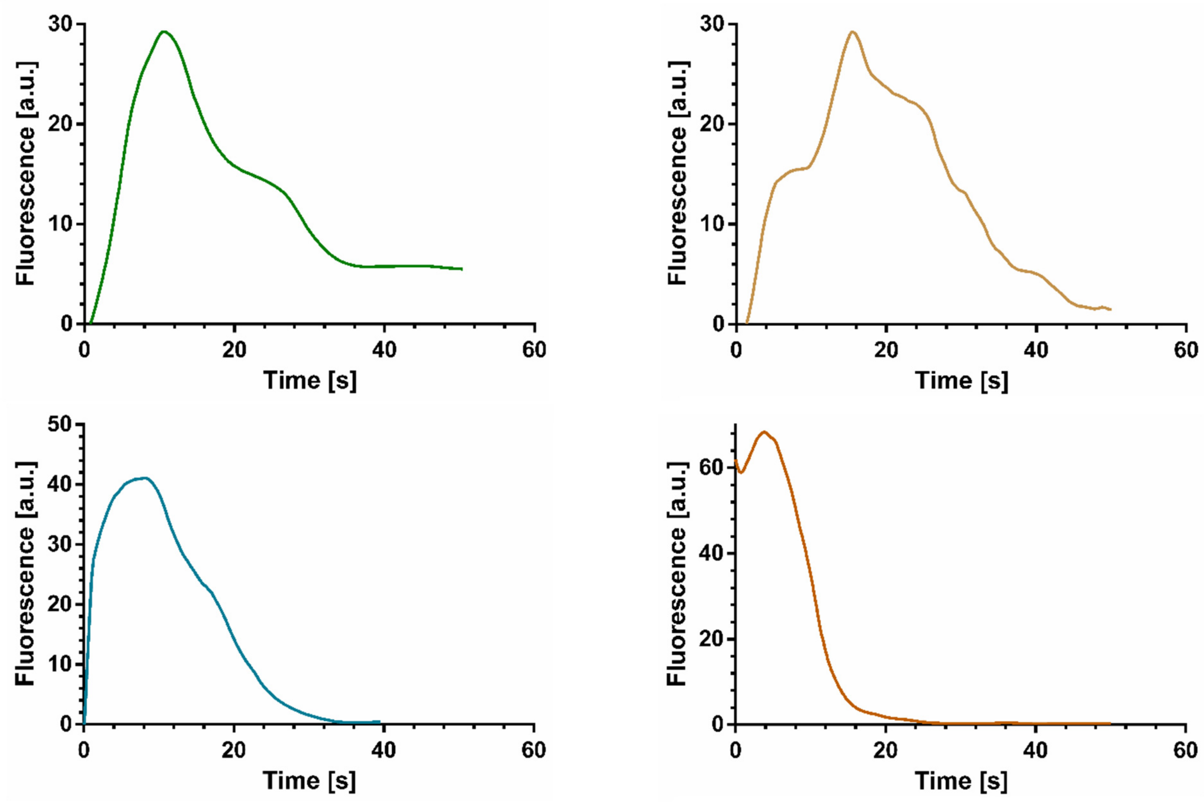

Figure S7. Worms fed with *E. coli* OP50 RFP. Fluorescence signal corresponding to the hindgut region for 4 representative worms. These signals were used to determine the average exponential decay curve in Figure 6f and to evaluate the absorption time constant τ of the digestive process in the worm intestine.

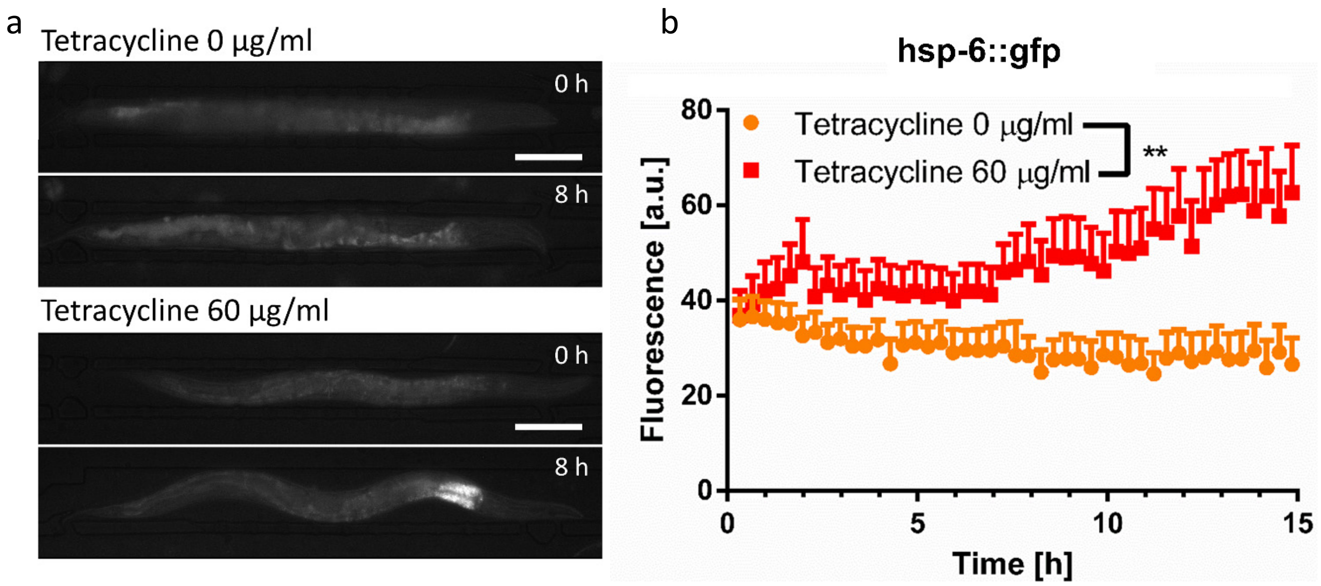

Figure S8. UPR^{mt} response in *C. elegans hsp-6::gfp* mutants induced by the antibiotic tetracycline. (**a**) Time-lapse images of immobilized YA *hsp-6::gfp* mutants, without and with tetracycline exposure. Scale bars = 100 µm. (**b**) Average of fluorescence signal related to *hsp-6::gfp* expression for treated and untreated worms. Error bars correspond to mean ± SD, ** $p \leq 0.01$, $n = 10$.
