# Supplementary material for: An In Vivo Microfluidic Study of Bacterial Load Dynamics and Absorption in the C. elegans Intestine
Source: Micromachines (Basel). 2021 Jul 17;12(7):832. doi: 10.3390/mi12070832 (PMC8304684; doi:10.3390/mi12070832)
Supplement: Supplementary file 1 [file micromachines-12-00832-s001.zip › micromachines-1293576-supplementary/ToC_f.pdf]

Table of Contents

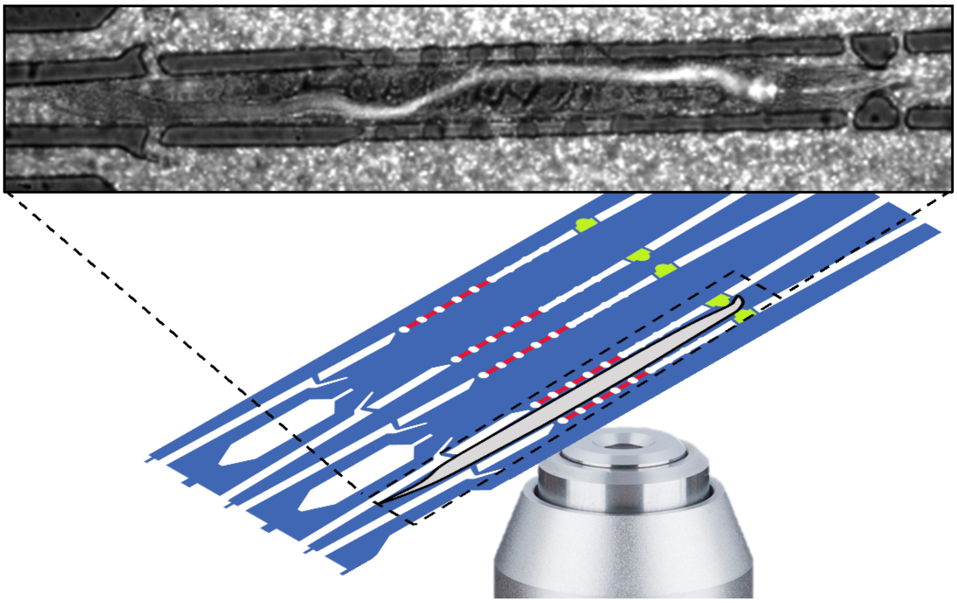

We present a microfluidic device to study *in vivo* bacterial load dynamics and food absorption in the intestine of *Caenorhabditis elegans*.
